# Aqueous microRNA profiling in age-related macular degeneration and polypoidal choroidal vasculopathy by next-generation sequencing

**DOI:** 10.1038/s41598-023-28385-7

**Published:** 2023-01-23

**Authors:** Yeong A. Choi, Areum Jeong, Chang-Hoon Woo, Soon Cheol Cha, Do Young Park, Min Sagong

**Affiliations:** 1 Department of Ophthalmology, Yeungnam University College of Medicine, #170 Hyunchungro, Nam-Gu, Daegu, 42415 South Korea; 2 Yeungnam Eye Center, Yeungnam University Hospital, Daegu, South Korea; 3 Department of Pharmacology, Yeungnam University College of Medicine, Daegu, South Korea

**Keywords:** Next-generation sequencing, Macular degeneration

## Abstract

Although many studies demonstrated the differences of clinical features, natural course, and response to treatment between typical age-related macular degeneration (AMD) and polypoidal choroidal vasculopathy (PCV), differential microRNAs (miRNAs) expression in the aqueous humor (AH) between them has not been reported yet. We investigated the roles of miRNAs in the AH of patients with typical AMD and PCV using next-generation sequencing (NGS) and quantitative PCR (qPCR). Target genes and predicted pathways of miRNAs were investigated via pathway enrichment analysis using the Kyoto Encyclopedia of Genes and Genomes database. A total of 161 miRNAs from eyes with typical AMD and 185 miRNAs from eyes with PCV were differentially expressed. 33 miRNAs were commonly upregulated, and 77 miRNAs were commonly downregulated in both typical AMD and PCV groups. Among them, hsa-miR-140-5p, hsa-miR-374c-3p, and hsa-miR-200a-5p were differentially expressed and were predicted to regulate proteoglycans in cancer, p53 signaling pathway, Hippo signaling pathway, and adherens junction. The differential expression profiles and target gene regulation networks of AH miRNAs may contribute to the development of different pathological phenotypes in typical AMD and PCV. The results of this study provide novel insights into the pathogenesis, associated prognostic biomarkers, and therapeutic targets in AMD and PCV.

## Introduction

Neovascular age-related macular degeneration (nAMD), which is characterized by choroidal neovascularization (CNV), accounts for most cases with loss of central vision due to AMD^1^. Polypoidal choroidal vasculopathy (PCV) is an exudative maculopathy characterized by abnormal aneurysmal dilatations and branching vascular network in the choroid^2^. Despite tremendous advancements in the characterization of the clinical features of typical AMD and PCV, little is known about the difference of pathophysiological mechanisms responsible for the development and progression between them^3–5^. To date, it is unknown whether PCV is a specific entity or a subtype of nAMD^3^.

MicroRNAs (miRNAs) are small (~ 19–22 nucleotides) endogenous non-coding RNAs that control gene expression through post-transcriptional regulation. Recent analyses have shown that certain miRNA species are expressed in all retinal cell types, while others are cell-type specific^4,5^. As miRNAs play important roles in biological processes, their dysregulation is associated with retinal degenerative diseases^6^. Recent technological advances have allowed miRNA expression profiling, and next-generation sequencing (NGS) methods have facilitated the application of big data to disease-associated genome-wide profiling^7,8^.

Several studies have reported that miRNAs derived from aqueous humor (AH) may be associated with ophthalmic diseases^9–11^. Differential expression of miRNAs was found in primary open-angle glaucoma eyes with varying degrees of visual field damage, and these differentially expressed miRNAs showed correlations with clinical features, thus indicating the possibility that miRNAs influence the glaucoma endophenotype^12^. Another study showed the expression profiles of AH-derived miRNAs and their potential roles in myopia development using bioinformatics analysis^13^. However, most of the previous studies investigated the expression of serum/plasma miRNAs in patients with nAMD^14^. Furthermore, differential miRNA expression between typical AMD and PCV has not been reported yet.

In this study, we compared differential miRNA expression in the AH of patients with typical AMD and PCV, as well as investigated miRNA profiles in each disease and miRNA-dependent mechanisms. The identification of all aberrantly expressed miRNAs and prediction of their functions using NGS shall provide a better understanding of the pathological processes in typical AMD and PCV.

## Results

### Demographics and baseline characteristics of patients

AH samples from 36 patients with typical AMD, 17 patients with PCV, and 18 control subjects with cataract were used. The mean ages of the control, typical AMD, and PCV groups were 65.28 ± 11.50, 69.75 ± 7.74, and 70.06 ± 8.15, respectively. Significant difference among 3 groups were noted in the refractive error (*p* = 0.015). There was no significant difference among 3 group in the mean intraocular pressure (IOP) (*p* = 0.314) (Table 1).

**Table 1:** Baseline demographics and characteristics of controls and patients with typical AMD and PCV.

	Control	Typical AMD	PCV	*p*-value
Number of eyes	18	36	17	
Age (year)	65.28 ± 11.50	69.75 ± 7.74	70.06 ± 8.15	0.178
Gender (male: female)	9:9	19:17	11:6	0.738
BCVA (logMAR)	0.68 ± 0.38	0.41 ± 0.44	0.74 ± 0.26	0.009
Refractive error (diopter)	+ 0.71 ± 0.68	− 0.13 ± 1.11	+ 0.43 ± 1.10	0.015
IOP (mmHg)	13.53 ± 2.85	16.50 ± 2.68	15.13 ± 4.20	0.352

Analysis of variance (ANOVA) followed by Bonferroni post hoc test (age, BCVA) and χ^2^ test (gender) were performed.

Values are presented as the mean ± standard deviation.

*AMD* age-related macular degeneration, *PCV* polypoidal choroidal vasculopathy, *BCVA* best-corrected visual acuity, *logMAR* logarithm of the minimum angle of resolution, *IOP* intraocular pressure.

### Overall miRNA expression in AH determined by NGS

**Figure 1:**
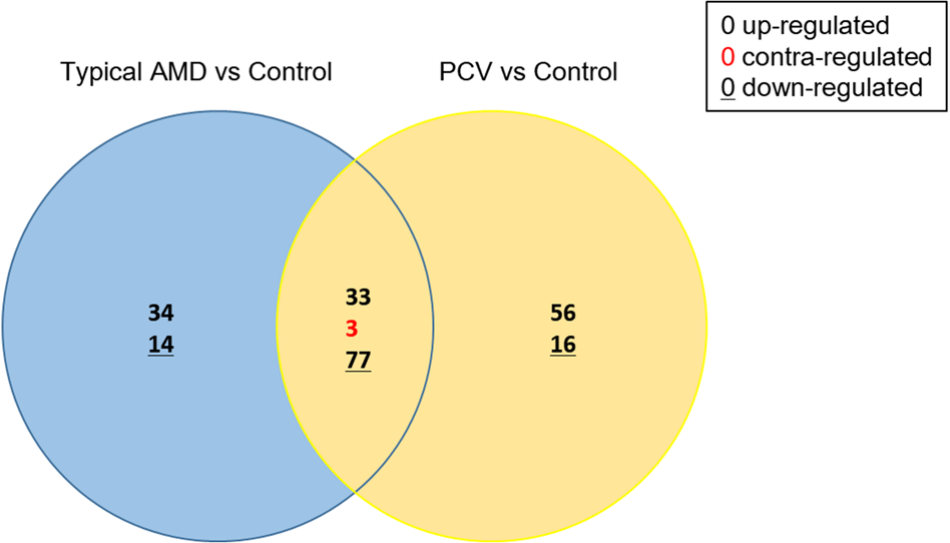
Venn diagram illustrating miRNAs detected in typical AMD and PCV eyes. *AMD* age-related macular degeneration, *PCV* polypoidal choroidal vasculopathy.

The mean RNA yield across all AH samples was 191.4–302.4 ng. A total of 161 miRNAs were detected in the typical AMD group, and a total of 185 miRNAs were detected in the PCV group compared to the control group. In total, 48 and 72 miRNAs were differentially expressed in typical AMD and PCV groups, respectively (Fig. 1).

### Identification of AH miRNAs in typical AMD and PCV groups

A total of 161 miRNAs, including 69 upregulated and 92 downregulated miRNAs, were expressed in the AH in the typical AMD group, as compared to the control group. Among the top 15 upregulated miRNAs, following miRNAs were selected and associated with typical AMD based on a PubMed search: hsa-miR-22-5p, hsa-miR-335-3p, hsa-miR-9-3p, hsa-miR-708-5p, hsa-miR-138-5p, and hsa-miR-20a-5p (Table 2).

**Table 2:** Top 15 upregulated miRNAs in typical AMD.

No.	microRNA	Fold change	Normalization data (log2)	
		Typical AMD/control	Typical AMD	Control
1	hsa-miR-22-5p	70.967	8.290	2.141
2	hsa-miR-502-3p	62.253	5.985	0.025
3	hsa-miR-335-3p	53.352	5.763	0.025
4	hsa-miR-499a-5p	48.852	5.635	0.025
5	hsa-miR-9-3p	35.811	6.267	1.105
6	hsa-miR-708-5p	29.918	4.927	0.024
7	hsa-miR-26a-2-3p	29.796	4.920	0.023
8	hsa-miR-138-5p	28.842	4.875	0.025
9	hsa-miR-1253	28.699	4.866	0.023
10	hsa-miR-20a-5p	26.584	4.757	0.024
11	hsa-miR-7706	24.396	4.634	0.025
12	hsa-miR-365a-3p	23.141	4.555	0.022
13	hsa-miR-330-3p	22.069	4.487	0.023
14	hsa-miR-424-5p	20.912	4.408	0.022
15	hsa-miR-181a-2-3p	19.953	4.344	0.025

*AMD* age-related macular degeneration.

A total of 185 miRNAs, including 90 unregulated and 95 downregulated miRNAs, were detected in the PCV group, as compared to the control group. Among the top 15 upregulated miRNAs, following upregulated miRNAs were selected: hsa-miR-374a-5p, hsa-miR-335-3p, hsa-miR-9-3p, hsa-miR-374b-5p, hsa-miR-20a-5p, and hsa-miR-22-5p (Table 3).

**Table 3:** Top 15 upregulated miRNAs in PCV.

No.	microRNA	Fold change	Normalization data (log2)	
		PCV/control	PCV	Control
1	hsa-miR-374a-5p	86.625	6.463	0.026
2	hsa-miR-335-3p	64.523	6.037	0.025
3	hsa-miR-509-3-5p	47.400	5.592	0.026
4	hsa-miR-181a-2-3p	42.188	5.424	0.025
5	hsa-miR-412-5p	40.196	5.355	0.026
6	hsa-miR-654-3p	37.924	5.269	0.024
7	hsa-miR-502-3p	35.650	5.181	0.025
8	hsa-miR-195-3p	32.200	5.032	0.023
9	hsa-miR-9-3p	31.732	6.092	1.105
10	hsa-miR-374b-5p	29.761	4.921	0.025
11	hsa-miR-20a-5p	29.304	4.897	0.024
12	hsa-miR-22-5p	29.047	7.001	2.141
13	hsa-miR-140-5p	28.508	6.547	1.714
14	hsa-miR-382-5p	26.612	4.759	0.025
15	hsa-miR-1298-5p	26.597	4.758	0.025

*PCV* polypoidal choroidal vasculopathy.

### Differential expression of miRNAs between typical AMD and PCV groups

Three differentially expressed miRNAs were selected based on their target genes. Compared to the typical AMD group, hsa-miR-140-5p, hsa-miR-374c-3p, and hsa-miR-200a-5p were upregulated in the PCV group (Table 4).

**Table 4:** Differentially expressed miRNAs in typical AMD-control, PCV-control, and typical AMD-PCV.

No.	miRNA	Fold change			Normalization data (log2)		
		Typical AMD/control	PCV/control	PCV/AMD	control	Typical AMD	PCV
1	hsa-miR-140-5p	0.310	28.508	91.826	1.714	0.027	6.547
2	hsa-miR-200a-5p	0.184	7.295	39.726	2.471	0.026	5.338
3	hsa-miR-374c-3p	0.473	15.947	33.702	1.106	0.026	5.101

*AMD* age-related macular degeneration, *PCV* polypoidal choroidal vasculopathy.

### miRNA validation by quantitative real-time PCR

**Figure 2:**
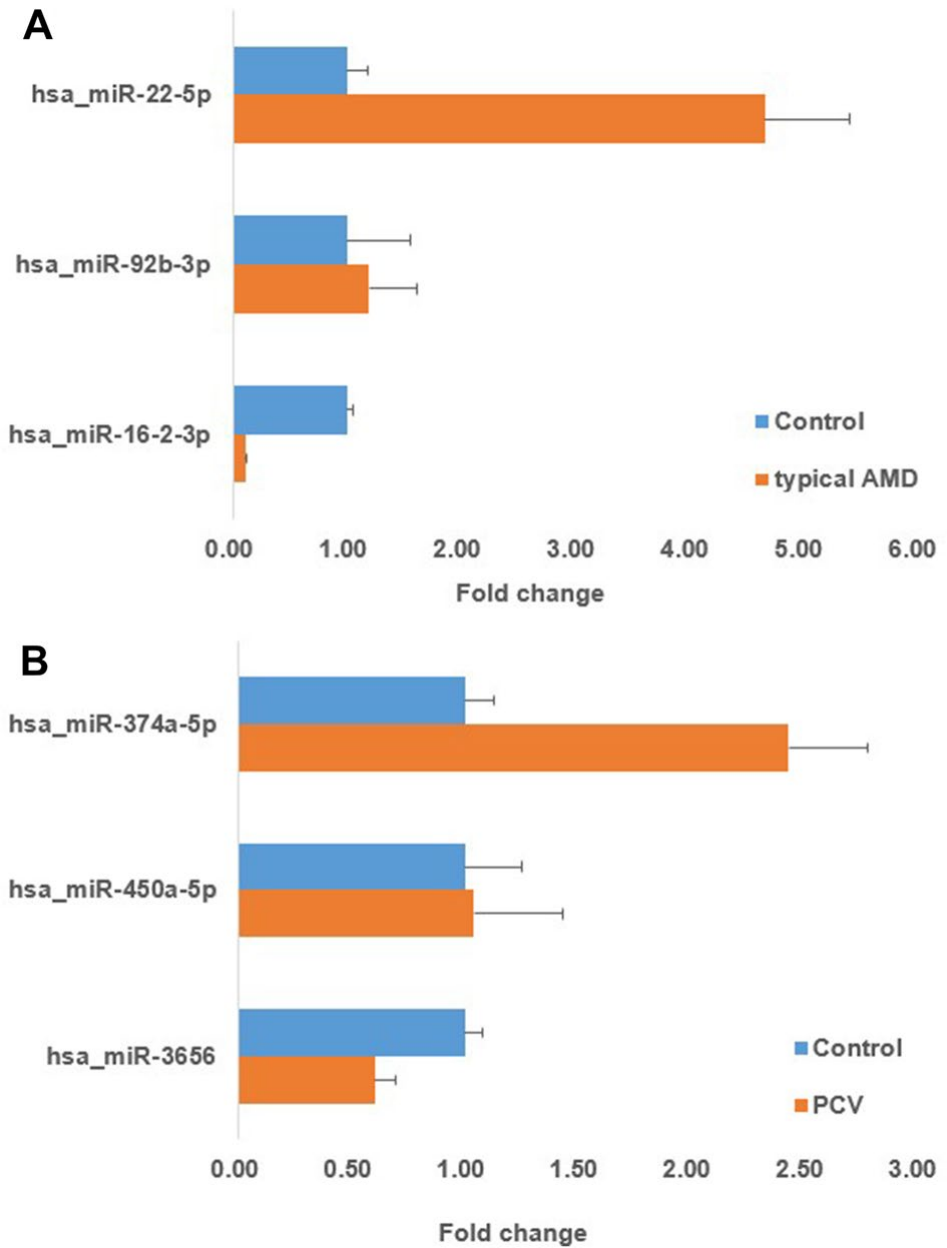
The relative expression levels of microRNAs (miRNAs) were assayed by quantitative PCR (qPCR) analysis and normalized to levels of A *Caenorhabditis elegans* miRNA-39 spike in control. The expression mode of three miRNAs of typical AMD (**A**) and three miRNAs of PCV (**B**) detected using qPCR. Error bars indicate standard deviations. *AMD* age-related macular degeneration, *PCV* polypoidal choroidal vasculopathy.

Based on current sequencing results, 6 miRNAs were selected: hsa-miR-22-5p in typical AMD and hsa-miR-374a-5p in PCV as high abundance miRNAs, hsa-miR-92b-3p in typical AMD and hsa-miR-150-5p in PCV as intermediate abundance miRNAs, and hsa-miR-16-2-3p in typical AMD and hsa-miR-3656 in PCV as low abundance miRNAs (Fig. 2).

### Analysis of target genes and their functions

**Figure 3:**
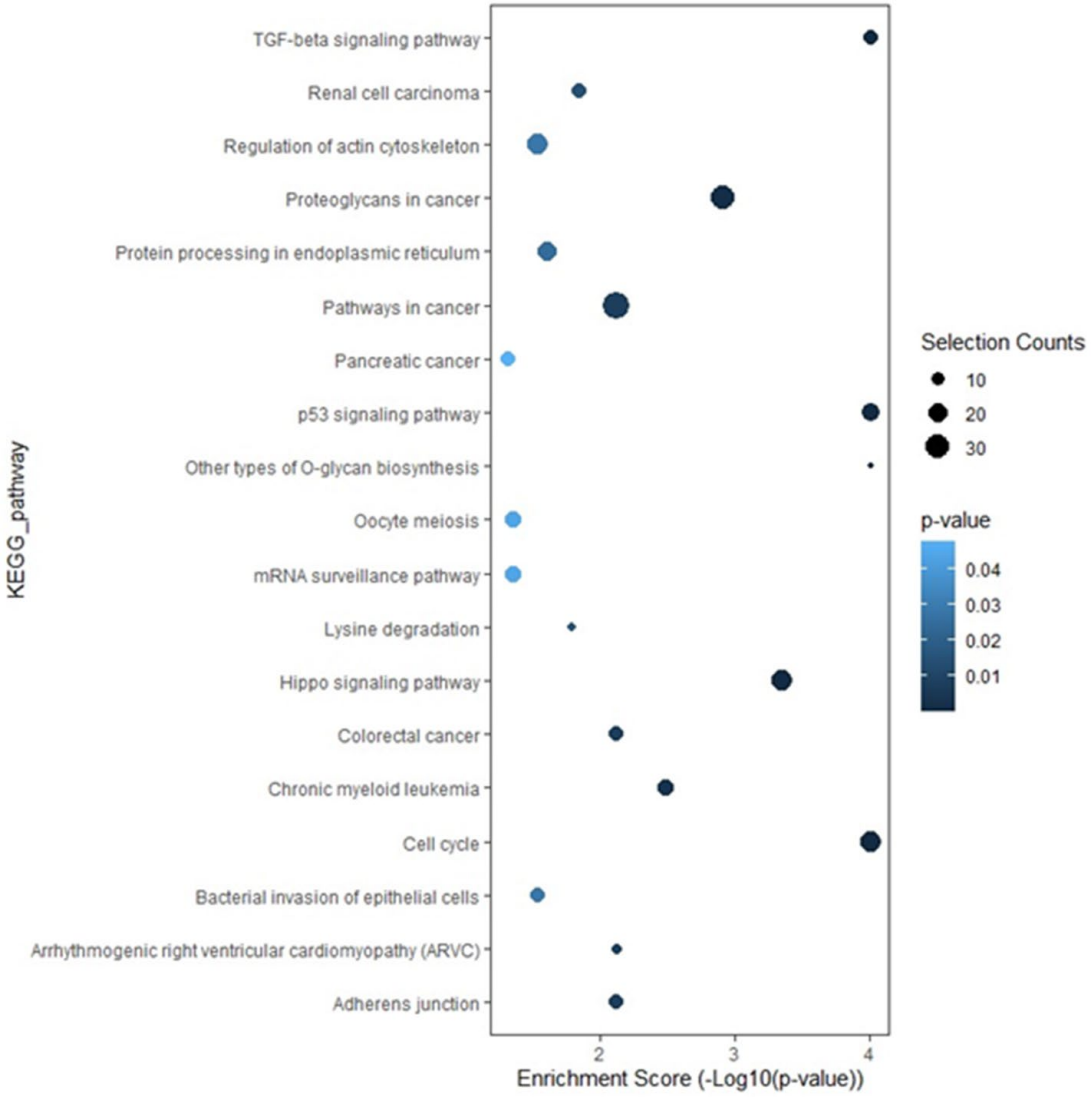
Kyoto Encyclopedia of Genes and Genome (KEGG) analysis of differentially expressed miRNAs between typical AMD and PCV eyes. *AMD* age-related macular degeneration, *PCV* polypoidal choroidal vasculopathy.

To investigate the various target genes of AH miRNAs, 6 upregulated miRNAs in typical AMD and PCV were selected for Kyoto Encyclopedia of Genes and Genomes (KEGG) enrichment analysis (see Supplementary Figs. S1 and S2 online). Three differentially expressed miRNAs of typical AMD and PCV were subjected to KEGG enrichment analysis (Fig. 3).

### Regulatory network of miRNA target genes

A miRNA target gene regulatory network was constructed. In the typical AMD group, 6 miRNAs formed a target gene regulatory network for the top 4 KEGG pathways: the Hippo signaling pathway, protein processing in endoplasmic reticulum (ER), proteoglycans (PGs) in cancer, and adherens junction (AJ) (see Supplementary Fig. S3 online). In the PCV group, 6 miRNAs were used to construct the target gene regulatory network for the top 4 KEGG pathways: the Hippo signaling pathway, TGF-β signaling pathway, PGs in cancer, and AJ (see Supplementary Fig. S4 online).

**Figure 4:**
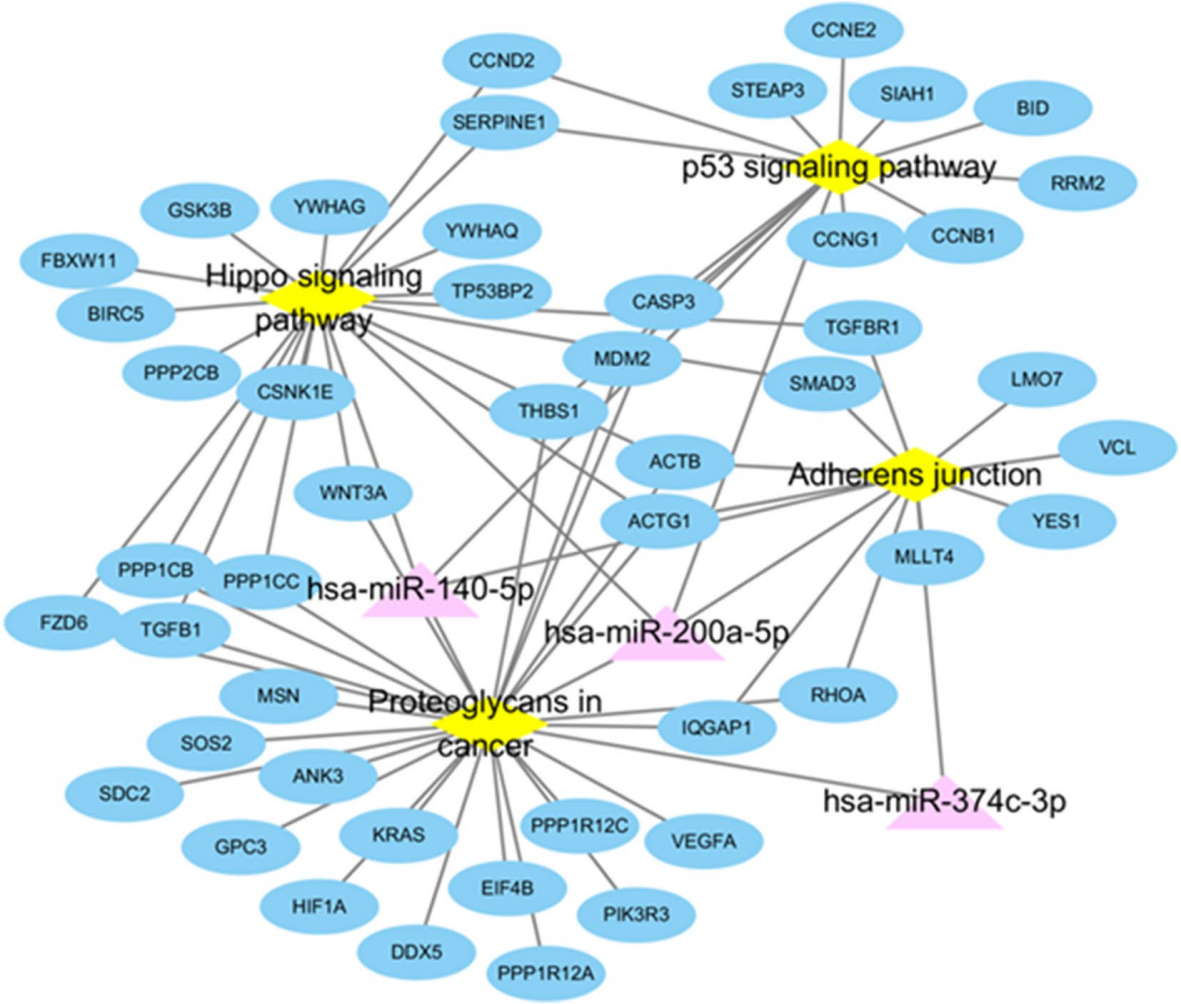
Predicted network of differentially expressed miRNAs (three by fold-changes in expression) between typical AMD and PCV eyes. *AMD* age-related macular degeneration, *PCV* polypoidal choroidal vasculopathy.

For comparison between the typical AMD and PCV groups, 3 differentially expressed miRNAs were analyzed to predict the target network. KEGG pathways (PGs in cancer, p53 signaling pathway, Hippo signaling pathway, and AJ) between the typical AMD and PCV groups were compared to predict the target network (Fig. 4).

## Discussion

In this study, we detected differentially expressed miRNAs in typical AMD and PCV and predicted the related pathways to understand the pathogenesis of typical AMD and PCV. Many different genetic strategies have been adopted to discover associated genes and variants for typical AMD and PCV^9^. Although several susceptible genes, such as complement factor H (*CFH)*, age-related maculopathy susceptibility 2 *(ARMS2)*, and high-temperature requirement factor H *(HTRA1)* have been identified in both typical AMD and PCV, genes such as *ARMS2-HTRA1* contribute to typical AMD susceptibility. Tanaka et al.^10^ reported that the *CFH* gene is more strongly associated with PCV than with typical AMD. Other studies have shown the expression of different cytokines in AH of typical AMD and PCV^11,15^. These differences supported the different pathogenic mechanisms between the two diseases^16^. Therefore, this study examined differentially expressed miRNAs that are associated with several pathogenic factors to understand the differences in the pathogenesis of typical AMD and PCV.

We compared published data on miRNAs of nAMD to our NGS data^14^. In previous studies, the upregulation of miR-9-3p in blood plasma^17^ and miR-20a-5p in serum^18^ were consistent with current data. Some studies have demonstrated that miR-9 plays a positive regulatory role in oxidative stress and inflammatory pathways and that miR-9 in the retinal pigment epithelium (RPE) cell line is overexpressed during apoptosis^19,20^. Moreover, miR-9 is associated with the nuclear factor-κB (NF-κB) signaling pathway and pathogenic mechanism of CFH deficiency, which leads to inflammatory neurodegeneration^21^. Thus, the upregulated miR-9-3p may play a crucial role in the inflammatory and oxidative mechanisms of typical AMD. It has been suggested that miR-20a levels in serum increase angiogenesis in patients with typical AMD and that miR-20a, which is upregulated under hypoxic conditions, targets VEGF^22,23^. In previous study, the downregulation of miR-335 in blood plasma was inconsistent with the current data that hsa-miR-335-3p was upregulated in the typical AMD groups^17^. These differences show that it might be difficult to infer the expression of ocular cell-specific miRNAs from that of miRNAs found in blood plasma. Activation of miR-335 plays an important role in the induction of p53-dependent cell cycle arrest after DNA damage^22^. p53 has been known to lead to RPE apoptosis in RPE-related aging disorders such as typical AMD. Taken together, these observations imply that miR-335 and miR-20a may play crucial roles in the apoptosis mechanism of typical AMD. In our study, hsa-miR-22-5p was the most abundant miRNA in the typical AMD group. miR-22 contributes to ischemic injury-induced mitochondrial oxidative damage and cell injury by targeting sirtuin-1, which may promote CNV^22,24^. In this study, hsa-miR-708-5p was overexpressed in the typical AMD group. miR-708-5p regulates rhodopsin in the retina and alleviates protein folding demand by reducing the flow of rhodopsin into the stressed ER^25^. Our results predict that the upregulation of miR-708-5p may relate to the oxidative stress pathway in typical AMD. In our study, hsa-miR-138-5p was highly expressed in typical AMD group. An experimental study showed that miR-138 induces exosome-mediated inflammation and apoptosis through the VEGF/NF-κB signaling pathway, which includes various biological processes, such as cell growth, cell differentiation, and inflammatory and immune responses^26^. The upregulation of hsa-miR-138-5p may be involved in the inflammatory pathogenesis of typical AMD.

Since, to our knowledge, there are no studies on the miRNA profiling of PCV, we compared the miRNA profiles of AMD from other studies with NGS data of PCV in this study. In the PCV group, the top 4 miRNAs related to angiogenesis (hsa-miR-374a-5p, hsa-miR-9-3p, hsa-miR-374b-5p, and hsa-miR-20a-5p) were selected. Additionally, hsa-miR-9-3p, hsa-miR-20a-5p, hsa-miR-335-3p, and hsa-miR-22-5p were commonly overexpressed in both typical AMD and PCV. This indicates that the overexpressed miRNAs in typical AMD and PCV may be involved in the common pathophysiology of typical AMD and PCV. In our study, hsa-miR-374a-5p was the most highly overexpressed miRNA in the PCV group and was one of the most abundant angiogenesis-related miRNAs. Bian et al.^27^ demonstrated that the targets of miR-374 family members mainly include the AKT, VEGF, PTEN, and Fas signaling pathways^27–29^. Tasharrofi et al.^30^ demonstrated that miR-374a inhibits Fas-induced apoptosis in human primary RPE by targeting Fas under oxidative conditions. This implies that oxidative conditions induce upregulation of miR-374a, leading to inhibition of Fas-induced apoptosis to protect RPE cells against oxidative stress in PCV. hsa-miR-374b-5p, is overexpressed in patients with PCV. Gutiérrez et al.^31^ demonstrated that upregulation of miR-374b leads to angiogenesis. Thus, miR-374b may contribute to angiogenic processes in PCV. Further in vivo and in vitro investigations of the miR-374 family are needed to confirm the miRNAs that may be associated with PCV. Taken together, these results suggest that the pathogenesis of PCV may be related to angiogenesis, oxidative stress, and inflammatory processes involving these miRNAs.

In this study, hsa-miR-140-5p, hsa-miR-200a-5p, and hsa-miR-374c-3p were upregulated in the PCV group but downregulated in the typical AMD group. hsa-miR-140-5p mediates the immune-related target genes and affects NF-κB signaling and the production of pro-inflammatory cytokines^32^. miR-140-5p suppresses the c-Met/AKT/mTOR signaling pathway in retinoblastoma cells and regulates various biological processes^33^. Sun et al.^34^ reported that miR-140-5p may directly target VEGFA and downregulate VEGFA protein expression. These findings suggest that miR-140-5p is associated with both angiogenesis and inflammatory mechanisms of typical AMD and PCV. Upregulated miR-140-5p in the PCV group was more closely related to the inflammatory pathway, whereas downregulation of miR-140-5p in the typical AMD group was more closely associated with the angiogenesis pathway. In a previous study, the miR-200 family inhibited angiogenesis by modulating the target gene *VEGF*, which promotes blood-retinal barrier breakdown and CNV^35^. Thus, the downregulation of hsa-miR-200a-5p may reflect the mechanism of angiogenesis in typical AMD. Yang et al.^23^ demonstrated that miR-374c regulates forkhead transcription factor 1, promotes proliferation and migration, and inhibits apoptosis in hypoxic condition. Therefore, this study suggests that the regulation of miR-347c may participate in cell proliferation, migration, and apoptosis in PCV and typical AMD. These findings indicate that typical AMD and PCV are both related to angiogenesis and inflammatory mechanisms, and differentially expressed miRNAs should be investigated to gain a better understanding of the pathophysiology of typical AMD and PCV.

miRNAs interact with target genes to regulate biological processes, and miRNA-associated algorithms provide the roles of miRNAs and their related target genes^36,37^. The 6 upregulated miRNAs in typical AMD regulate protein processing in the ER, the Hippo signaling pathway, the AJ, and PGs in cancer. The 6 upregulated miRNAs in the PCV group regulate the Hippo signaling pathway, PGs in cancer, the TGF-β signaling pathway, the AJ, and protein processing in the ER. The current results indicate that miRNAs showing differential expression profiles in both typical AMD and PCV groups regulate common pathways. The Hippo pathway regulates endothelial cell proliferation, migration, and the major intracellular signaling pathways that regulate angiogenesis^38^. ER stress-mediated VEGF expression is involved in the process of angiogenesis^28^. The TGF-β signaling pathway regulates the expression of molecules related to inflammation, vascular fibrosis, and angiogenesis^29^. PGs play a role in extracellular matrix deposition in the Bruch's membrane thickening and accelerate neovascularization in AMD^39^. Damage to the integrity of the AJ in the RPE contributes to the p53/MDM2 pathway and disrupts photoreceptor homeostasis in the apoptotic and inflammatory processes^40^. Thus, the results of pathway and gene target analyses indicated that typical AMD and PCV share a common pathogenesis, and differentially expressed miRNAs may participate in the pathogenesis of the two diseases.

In the present study, 3 miRNAs showed significant differential expression between the PCV and typical AMD groups. These miRNAs were predicted to be involved in PGs in cancer, p53 signaling pathway, Hippo signaling pathway, and AJ in the pathogenesis and progression of PCV and typical AMD. Under oxidative stress or during aging, the p53 signaling pathway leads to decreased MDM2 binding and consequently triggers RPE apoptosis^40^. In this way, the dysregulation of 3 miRNAs related to the p53 signaling pathway may shed light on the pathogenesis of typical AMD and PCV.

This study had several limitations. First, the sample size was relatively small, and this study included individuals with cataract eyes as the control group. Despite a lack of healthy aqueous humor donors, the aqueous humor of cataract eyes has been commonly used as a source of normal controls in previous studies. Second, the identified miRNAs in this study had both similarities and differences with those from other AMD studies. Because of inconsistencies in patient inclusion criteria or exclusion criteria, different analytical methods used to quantify, or selection of appropriate controls, different miRNA profiles were obtained^14^. Future studies should focus on well-identified miRNA profiles. Third, this study only evaluated miRNA profiles in the AH and not in the vitreous humor or peripheral blood, which could have revealed the relationship between local and systemic changes in miRNA profiles. Fourth, we only identified specific miRNAs by NGS and predicted the target genes. Additionally, this study did not investigate whether differentially expressed miRNAs affect the expression of proteins or cytokines.

In conclusion, the differential expression profiles of miRNAs and their target gene regulatory networks provide new insights into molecular pathogenetic mechanisms and useful information for the development of novel diagnostic biomarkers and the identification of therapeutic targets for typical AMD and PCV.

## Methods

### Subjects

The study protocol was approved by the Institutional Review Board of Yeungnam University Hospital (IRB No.: 2020-04-129-002). The study was performed in accordance with the tenets of the Declaration of Helsinki. The study population consisted of 18 control subjects who had undergone cataract surgery, 36 patients with treatment naïve-typical AMD, and 17 patients with treatment naïve-PCV. Patients with typical AMD and PCV were diagnosed by a retinal specialist (MS). The typical AMD was diagnosed based on the presence of CNV, while PCV was diagnosed using EVEREST study criteria^41^. The exclusion criteria were as follows: age < 50 years, prevalence of eye diseases other than typical AMD and PCV, prior ocular surgery, laser photocoagulation, or intraocular injection of anti-VEGF or steroids; history of other systemic diseases. Written informed consent was obtained from all patients for the collection of AH and scientific use of the specimen.

### Aqueous humor sampling and RNA extraction

A 50–100 μL of AH was collected at the outsets of cataract surgery of control eyes and intravitreal injection of typical AMD and PCV eyes. AH sample was acquired by paracentesis using a 30-gauge needle under aseptic conditions. The AH was acquired without trauma or any possibility of contamination of cellular debris. All samples were immediately stored at − 80 °C and transported to laboratories. The RNA was extracted from 200 μL of each AH sample with minor modifications. TRIzol LS reagent (Invitrogen, Carlsbad, CA, USA) was added to the AH samples prior to purifying with the column. The quantity of the obtained miRNA was measured using a NanoDrop 2000 spectrophotometer (Thermo Fisher Scientific, Waltham, MA, USA). All AH samples were pooled by group before sequencing.

### Library preparation and NGS sequencing data analysis

RNA-sequencing libraries were constructed employing a NEBNext Multiplex Small RNA Library Prep kit (New England BioLabs, Ipswich, MA, USA) with a 6 μL of pooled sample. And 10 μL of pooled sample was ligated with specific adaptors, and cDNA was synthesized using reverse transcriptase with adaptor-specific primers. The libraries amplified by PCR were cleaned using the QIAquick PCR Purification Kit (Qiagen, Düsseldorf, Germany) and AMPure XP beads (Beckman Coulter, Pasadena, CA, USA). The yield and size distribution of the libraries were determined using an Agilent 2100 Bioanalyzer for a high-sensitivity DNA assay (Agilent Technologies). High-throughput sequencing was performed using a NextSeq500 system with single-end 75 sequencing (Illumina, San Diego, CA, USA).

To obtain alignment files (BAM files), sequencing reads were mapped using Bowtie2^42^. Mature miRNA sequencing was performed to obtain a reference for mapping. Read counts were used to determine the AH miRNA expression levels. Mature miRNA sequences were extracted from the alignment file using BEDTools (v2.25.0)^43^. Bioconductor (EdgeR package) that employed R statistical programming language (R Foundation for Statistical Computing, Vienna, Austria, version 3.2.2) was used for normalization. It normalized sequencing reads by using the TMM (trimmed mean of m-values) method and filters using a nominal, user-defined CPM (counts-per-million). Differentially expressed miRNAs that showed a 2.0-fold change in their expression level were selected and compared among the typical AMD, PCV, and control groups. Data mining and graphic visualization were performed using ExDEGA (Ebiogen Inc., Seoul, South Korea).

### miRNA validation by quantitative real-time PCR

RNA samples were precipitated by centrifugation (13,000 rpm for 10 min at 4 °C). The supernatant was removed, and the RNA pellet was washed with 1 mL of 70% ethanol. RNA yield was determined based on the absorbance at 160 nm (A_160_), and RNA purity was assessed using a NanoDrop 2000 Spectrophotometer (Thermo Fisher Scientific). Reverse transcription was performed using the miRCURY LNA RT Kit (Qiagen, Cat No. 339340). Real-time qPCR was performed on a StepOnePlus™ Real-Time PCR System (Thermo Fisher Scientific) using a miScript SYBR Green PCR Kit (Qiagen, Düsseldorf, Germany) according to the manufacturer’s instructions. Quantification was performed using StepOne software (v2.2.2; Thermo Fisher Scientific, Waltham, MA, USA).

### Bioinformatics and statistical analyses

The KEGG database was used to evaluate biological pathways and to screen target genes through the online miRPath 3.0 using miRWalk^22^. KEGG pathway enrichment analysis revealed the predicted target genes associated with typical AMD and PCV^44,45^.

Clinical data were analyzed using IBM SPSS (version 25 for Windows; IBM Corp., Chicago, IL, USA). The χ^2^ test and Fisher’s exact test were used to analyze differences in the gender ratio between the groups. Analysis of variance (ANOVA) was used to compare age, best-corrected visual acuity, IOP, and refractive error among the three groups. Expression levels of miRNAs in typical AMD and PCV samples normalized relative to the AH of cataract controls were compared using the two-tailed *t-test* (control vs. typical AMD, control vs. PCV). Moreover, levels of miRNA expression between typical AMD and PCV were compared using a two-tailed t-test (typical AMD vs. PCV). In all analyses, statistical significance was set at *p* < 0.05.

## Supplementary Information

Supplementary Figure S1.

Supplementary Figure S2.

Supplementary Figure S3.

Supplementary Figure S4.

## Data Availability

The datasets generated and analyzed during the current study are available in the Gene Expression Omnibus (GEO) repository, Accession number to datasets: GSE221905.
