# Supplementary figures and images for: Aqueous microRNA profiling in age-related macular degeneration and polypoidal choroidal vasculopathy by next-generation sequencing
Source: Sci Rep. 2023 Jan 23;13:1274. doi: 10.1038/s41598-023-28385-7 (PMC9870898; doi:10.1038/s41598-023-28385-7)

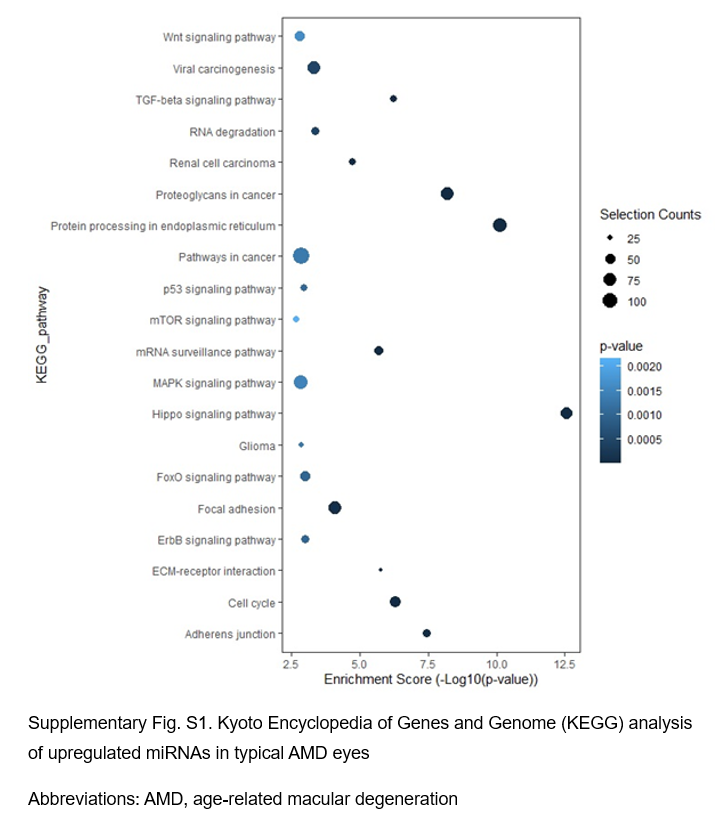

Supplement: Supplementary file 1 — Supplementary Figure S1. [file 41598_2023_28385_MOESM1_ESM.tif]

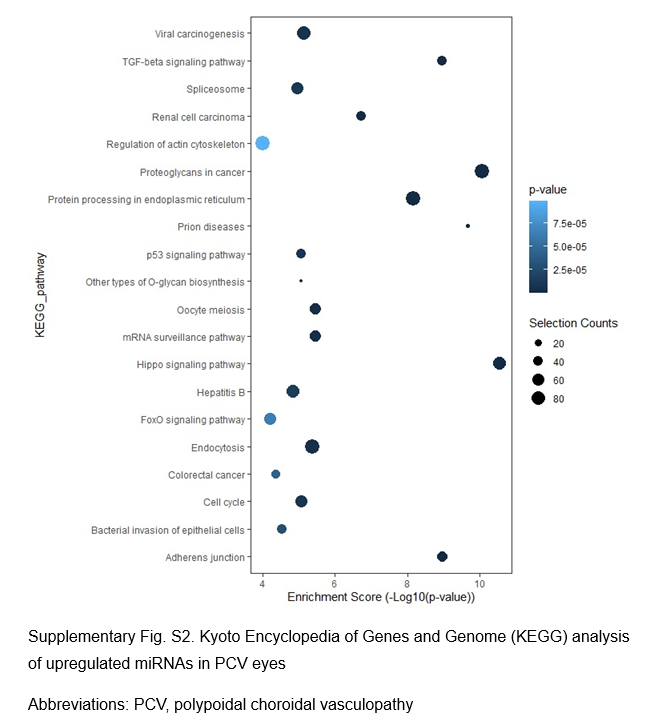

Supplement: Supplementary file 2 — Supplementary Figure S2. [file 41598_2023_28385_MOESM2_ESM.tif]

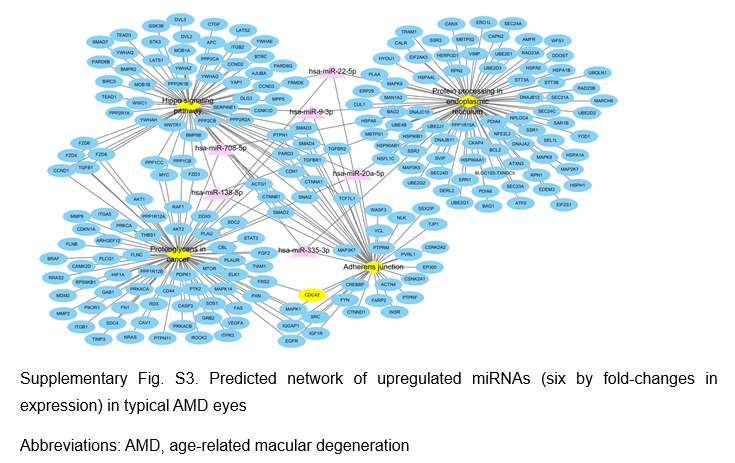

Supplement: Supplementary file 3 — Supplementary Figure S3. [file 41598_2023_28385_MOESM3_ESM.tif]

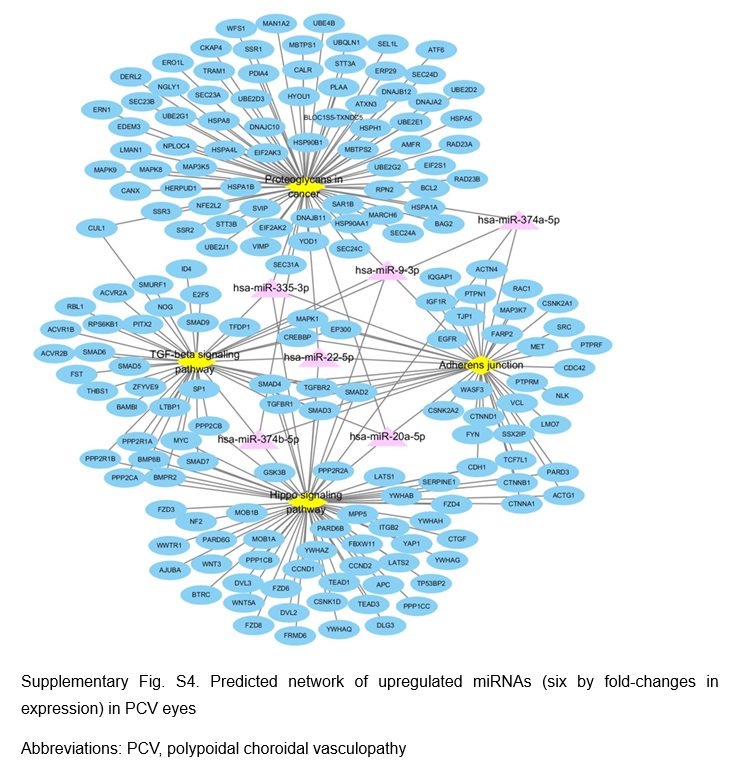

Supplement: Supplementary file 4 — Supplementary Figure S4. [file 41598_2023_28385_MOESM4_ESM.tif]
